# Prospective study on retinal nerve fibre layer changes after an acute episode of phacomorphic angle closure

**DOI:** 10.1007/s10792-012-9614-5

**Published:** 2012-07-31

**Authors:** Jacky W. Y. Lee, Jimmy S. M. Lai, Doris W. F. Yick, Can Y. F. Yuen

**Affiliations:** 1 The Eye Institute, The University of Hong Kong, Room 301, Level 3, Block B, Cyberport 4, 100 Cyberport Road, Hong Kong, SAR People’s Republic of China; 2 The Department of Ophthalmology, Caritas Medical Centre, Hong Kong, SAR People’s Republic of China

**Keywords:** Retinal nerve fibre layer, Optical coherence tomography, Phacomorphic, Cup-disc ratio, Humphrey visual field

## Abstract

To investigate the retinal nerve fibre layer (RNFL) changes after an acute attack of phacomorphic angle closure. This prospective study involved ten cases of phacomorphic angle closure that underwent cataract extraction and intraocular lens insertion after intraocular pressure lowering. Apart from visual acuity and intraocular pressure (IOP), RNFL thickness and vertical cup disc ratio (VCDR) were measured by optical coherence tomography (OCT) at 3–9 months post attack. Humphrey visual field assessment was performed at 9 months post attack. All cases had mean phacomorphic duration of <5 days. Postoperatively, best correct Snellen visual acuity was 0.4 ± 0.2 and IOP at 9 months was 11.0 ± 3.1 mmHg. There was no difference in VCDR and RNFL between the attack and contralateral eye at 3 months post attack (both *p* = 0.4). At 9 months post attack, there was significant thinning in the average (*p* = 0.01), superior (*p* = 0.01), and inferior (*p* = 0.006) RNFL. There was no significant difference in the pattern standard deviation (PSD) between the two eyes on the Humphrey visual field nor was there any correlation between PSD severity and RNFL thinning (all *p* > 0.2. Patients with <5 days duration of phacomorphic angle closure are likely to have reasonable postoperative vision. An acute episode of phacomorphic angle closure can trigger an accelerated RNFL thinning despite normal IOP and open angles, most noticeable in the superior and inferior quadrants, occurring between 3 and 9 months post attack. Glaucomatous optic neuropathy in the attack eye was evident by OCT but not by visual field assessment at the same time interval.

## Introduction

Phacomorphic angle closure is a secondary angle closure caused by a swollen and mature cataract obstructing the drainage angle, leading to an acute elevation of intraocular pressure (IOP) and potential glaucomatous optic neuropathy (GON) if not treated timely. The initial aim of treatment of phacomorphic angle closure is to lower the IOP with combinations of topical anti-glaucoma medications, systemic acetazolamide, intravenous mannitol and or argon laser iridoplasty (ALPI) [1]. All of these treatments have been established to be effective initial treatments with no evidence showing superiority of one over the other at the moment. The definitive treatment after IOP control is cataract extraction.

The term phacomorphic glaucoma is used if there resultant GON [1] which has been previously quantified and reported using visual field (VF) assessments or clinical cup-disc ratio monitoring. However, both of these parameters are variable and not entirely objective especially when phacomorphic angle closure often affects an elderly population where dementia and neglect are common [2]. In addition, studies in acute primary angle closure (APAC) have shown that more than half of the patients with a single attack can have no visual field defects [3]. On the other hand, retinal nerve fiber layer measurements can detect early GON as its damage often precedes visual field loss [4]. The use of optical coherence tomography (OCT) for retinal nerve fibre layer (RNFL) and optic nerve head analyses is non-invasive, requires minimal patient cooperation, and provides objective and early assessment of GON in patients with phacomorphic angle closure.

## Purpose

To investigate the RNFL changes after an acute attack of phacomorphic angle closure.

## Patients and methods

Consecutive cases of acute phacomorphic angle closure from December 2009 to December 2010 were recruited from Caritas Medical Center, Hong Kong Special Administrative Region, People’s Republic of China. Patient’s intraocular pressure was lowered initially either by ALPI or systemic acetazolamide. The selection between the two initial treatments was randomized. The randomization was part of a treatment protocol of another study comparing the effects of initial treatments in phacomorphic angle closure. Those receiving ALPI received laser applications 360° to the peripheral iris with power titrated to achieve visualized contractions of the iris. All ALPI was performed by a single surgeon (JL). Those receiving systemic acetazolamide received intravenous acetazolamide 500 mg stat followed by oral acetazolamide 250 mg four times daily and slow-release potassium chloride tablets 600 mg twice daily if there were no systemic contraindications. All patients were put on the following eye drops: Atropine 1 % daily (Alcon Inc., Hünenberg, CH-6331, Switzerland), Pred forte1% four times daily (Allergan Inc., Irvine, CA 92623-9534, USA), and Timolol 0.5 % twice daily (Santen Pharmaceutical Co. Ltd., Osaka, 533-8651, Japan) in the attack eye. Patients with presenting IOP higher than 60 mmHg or IOP higher than 40 mmHg after 2 h of treatment were given 200 ml of 20 % mannitol intravenously over 1 h. Hourly IOP was documented until it was below 25 mmHg.

Cases were included for consenting individuals with a first episode of acute (<14 days) phacomorphic angle closure with an IOP more than 40 mmHg. The diagnosis of phacomorphic angle closure was based on the presence of an intumescent cataract and signs of acute angle closure: conjunctival injection, shallow anterior chamber, corneal edema in the index eye or an open angle in the contralateral eye as determined by gonioscopy [2]. Cases were excluded if IOP lowering treatment was given for this acute episode prior to the study or where ALPI was not possible either due to a severely edematous cornea or uncooperative patient.

All cases received extracapsular cataract extraction (ECCE) and intraocular lens (IOL) insertion under regional anesthesia by one of the three glaucoma surgeons at our center within 3 days of presentation to our clinic. All glaucoma medications were discontinued immediately after the cataract extraction. ECCE was chosen over phacoemulsification due to less endothelial damage from ultrasound energy and phacoemulsification would impose greater surgical risk in the setting of phacomorphic angle closure especially in the presence of any residual corneal edema and zonule loosening [2].

Patients were followed up on day one, 1 week, 1 month, 3 months and as needed postoperatively and IOP was measured by Goldman applanation tonometry in each visit. Best corrected visual acuity (BCVA) was measured by Snellen chart at 1 month postoperatively. The trabecular-iris angle was measured by gonioscopy and ultrasound biomicroscopy (UBM) at 3 months post attack.

Stratus optical coherence tomography (Carl Zeiss Meditec Inc., Dublin, CA, USA) was used for RNFL in micrometers (μm) and vertical cup-disc ratio (VCDR) measurements in both eyes at 3 and 9 months post phacomorphic attack. The pupils were pharmacologically dilated to at least 5 mm prior to OCT and all scans were performed using fast scan mode by a single operator (JL) with 4 years of operating experience. All scans had centered alignment and signal strength of six or more, suboptimal scans were repeated. Multiple scans (two to four per eye) were obtained per secession and the scan with the highest quality (highest signal strength and most centred alignment) was selected.

Visual field was assessed at 9 months post cataract extraction with the Humphrey Field Analyzer (Carl Zeiss Ophthalmic Systems, Inc., Dublin, CA, USA) using Swedish interactive threshold algorithm fast (SITA Fast), 24-2 strategy.

### Statistics

Paired Wilcoxon matched pairs (signed rank) test was used to compare the RNFL in the following settings:

1. Between the eye with phacomorphic angle closure and the contralateral (non-attack) eyes at 3 months.
2. Between the eye with phacomorphic angle closure at 3 and 9 months.
3. Between the contralateral eye at 3 and 9 months.

For the above tests, the four quadrants and average thickness were compared independently in a planned comparison basis.

Mann–Whitney *U* test was used to compare the RNFL and VCDR between eye with phacomorphic angle closure and contralateral eye as well as to compare the pattern standard deviation (PSD) on the VF between the two eyes. Pearson’s correlation was used to determine correlation between RNFL and PSD.

For all statistical calculations *p* < 0.05 was considered significant and all means were presented as mean ± standard deviation.

The institutional review board of the Hospital Authority of Hong Kong approved this study. The study protocol followed the principles in the Declaration of Helsinki. Informed consent was obtained from all patients prior to all treatments and investigations. The authors declare no proprietary interest or financial sponsorship.

## Results

Eleven consecutive cases of acute phacomorphic angle closure presented to our center during the study period. One case was later excluded because of defaulting follow-up after cataract extraction. Four cases received ALPI and six cases received systemic acetazolamide as the initial means of control IOP. All IOP’s were lowered to <25 mmHg within 5 h of treatment with corneal clarity achieved day one after treatment. Two cases received intravenous mannitol because one presented with IOP more than 60 mmHg and the other had persistent IOP >40 mmHg 2 h after treatment with systemic acetazolamide. There were no systemic side effects of mannitol. All cases received ECCE + IOL within 3 days of presentation to our center. There were no intra-operative complications. One case required scleral fixation of the IOL due to postoperative posterior chamber IOL subluxation 1 week after the initial operation. None of the cases had any documented history of ocular trauma.

For the ten cases, the mean age was 79.1 ± 8.3 years. The presenting IOP was 50.5 ± 7.4 mmHg. The mean time taken from phacomorphic symptoms to ophthalmic consultation was 2.1 ± 2.8 days (range: 0.5–10 days). The postoperative IOP at 3 months was 10.5 ± 3.4 mmHg without any glaucoma medication. At 9 months post operation, the mean IOP was 11.0 ± 3.1 mmHg; all except one patient required topical glaucoma medications for IOP control <21 mmHg. All cases had an open trabecular-iris angle configuration on gonioscopy and UBM.

The presenting mode visual acuity was light perception (LP). The BCVA postoperative VA was 0.4 ± 0.2, including a case was a pre-existing atrophic macula that had a presenting VA of LP and a postoperative VA of 0.1. There was no significant difference between the VCDR as measured by OCT in the attack (0.6 ± 0.2) and contralateral eye (0.6 ± 0.1) (*p* = 0.4).

At 3 months post attack, there was no significant difference between the average RNFL of the eye with phacomorphic angle closure (95.1 ± 21.5 μm) and the contralateral eyes (105.9 ± 27.8 μm) (*p* = 1.0). Analyses of each of the individual quadrants again showed no difference between the two eyes (all *p* > 0.2).

However, at 9 months post attack, the average RNFL of the phacomorphic eye had progressive thinning from 95.1 ± 21.5 μm at 3 months to 85.4 ± 21.0 μm at 9 months (*p* = 0.01). Quadrant analyses showed significant thinning in the superior (110.5 ± 32.74 to 95.9 ± 30.6 μm, *p* = 0.01) and inferior quadrants (117.8 ± 30.7 to 90.60 ± 25.5 μm, *p* = 0.006) but there was no significant progressive thinning in the nasal (*p* = 0.6) or temporal (*p* = 1.0) quadrants (Tables 1, 2).

**Table 1:** RNFL thickness (μm) of the phacomorphic eye versus contralateral eye at 3 months post attack

	Phacomorphic eye	Contralateral eye	*p* value
Superior quadrant	110.5 ± 32.7	121.5 ± 34.6	0.6
Nasal quadrant	71.1 ± 24.9	78.7 ± 24.7	0.3
Inferior quadrant	117.8 ± 30.4	132.9 ± 23.3	0.2
Temporal quadrant	76.3 ± 19.5	73.1 ± 14.4	1.0
Average thickness	94.4 ± 22.6	102.6 ± 20.3	1.0

**Table 2:** RNFL thickness (μm) in the phacomorphic and contralateral eyes at 3 versus 9 months post attack

	Contralateral eye at 3 months	Contralateral eye at 9 months	*p* value	Phacomorphic eye at 3 months	Phacomorphic eye at 9 months	*p* value
Superior quadrant	121.5 ± 34.6	115.8 ± 28.7	0.2	110.5 ± 32.7	95.9 ± 30.7	0.01*
Nasal quadrant	78.7 ± 20.7	76.7 ± 15.8	0.5	71.1 ± 24.9	74.0 ± 20.2	0.6
Inferior quadrant	132.9 ± 23.1	114.5 ± 31.2	0.4	117.8 ± 30.7	90.6 ± 25.5	0.006*
Temporal quadrant	73.1 ± 14.4	91.9 ± 37.4	0.3	76.3 ± 19.5	80.7 ± 34.0	1.0
Average thickness	102.6 ± 20.3	99.76 ± 17.8	0.1	94.4 ± 22.6	85.4 ± 21.0	0.01*

* Statistically significant (*p* < 0.05)

For the contralateral eye, there was no significant difference in the average RNFL (*p* = 0.1) nor individual quadrants from 3 to 9 months (all *p* > 0.2).

Six cases were able to produce reliable Humphrey visual field assessments. The other cases had poor fixation, unreliable parameters or defaulted the appointment. The mean deviation (MD) was −5.8 ± 3.7 decibels (db) and the PSD was 4.2 ± 3.0 db. There was no significant difference in the PSD between the attack (4.2 ± 3.0 db) and contralateral eye (3.1 ± 1.1 db) (*p* = 0.7). There was also no significant correlation between the RNFL thickness on OCT and the PSD on VF (*p* = 0.2).

## Discussion

In the literature, OCT for RNFL has been used to evaluate the damage after a single episode of acute primary angle closure and to offer a more objective supplement to the information provided by Humphrey visual field assessments. To the best of our knowledge, this is the first prospective clinical trial using OCT to investigate RNFL thickness and VCDR in phacomorphic angle closure.

Various studies have associated poor visual outcomes with a longer duration of phacomorphic symptoms. Lee et al. [2] have found a linear relationship between a longer duration of attack and a poorer VA. Ramakrishanan et al. [5] found that 70 % of their phacomorphic glaucoma patients had final BCVA of 20/40 when the duration of symptoms was <10 days. Similarly, Prajna et al. [6] found that 61 % of their phacomorphic patients had final visual acuity of 6/12 or better but that a glaucomatous process for more than 5 days was associated with poorer visual outcome. Our study was consistent with these finding in that our patients had a final mean BCVA of 0.4 ± 0.2; their mean phacomorphic symptom duration was 2 days and all received definitive cataract extraction within 3 days of presentation, resulting in a mean duration of phacomorphic angle closure of <5 days.

We have demonstrated that despite the definitive removal of the intumescent cataract, glaucomatous optic neuropathy was an ongoing progress that took place between 3 and 9 months after the initial attack. There was no significant RNFL difference between the phacomorphic and contralateral eye at 3 months but a significant thinning of the average, superior and inferior RNFL was observed at 9 months post attack in the eye with previous phacomorphic angle closure. The contralateral eye, on the other hand, showed no interval differences in the average or quadrant RNFL thicknesses.

Studies involving APAC have used scanning laser polarimetry to demonstrate that RNFL can be thinned after the attack [7, 8]. But no study to date can determine the rate of RFNL thinning after an episode of APAC [9, 10]. Aung et al. [7] has found that RNFL can decrease from anywhere between 2 and 16 weeks after a primary angle closure attack. Similarly, Tsai et al. [12] found no significant difference in RNFL thinning between 4 and 12 weeks post APAC but significant differences between 1 and 4, and 1 and 12 weeks. In fact, they found that the RNFL was thicker at 1 week in the eye with APAC compared the contralateral eye followed by a rapid thinning by 4 weeks, likely due to an initial optic nerve edema.

One may argue that the progressive RNFL thinning in primary acute angle closure may not all be attributed to the acute rise in IOP as this group of patients will have had narrow drainage angles for a certain duration and the RNFL thinning may represent damage from previous intermittent angle closures or a chronic angle closure in evolution. On the other hand, in phacomorphic angle closure, the pathology is in the sudden anterior bulging of the swollen cataract, thus, intermittent or asymptomatic chronic angle closure is less likely than in APAC. Thus, the GON that allows a phacomorphic attack is possibly mainly attributed to the acute IOP rise. Yoles and Schwartz [13] have suggested that GON progression can occur even after elimination of the acute rise in IOP as a result of secondary apoptosis of healthy neurons bathed in the degenerative milieu created by the damaged neurons from the acute event.

The other possibility for GON progression is a secondary angle closure that develops after the phacomorphic attack but all our cases had IOP < 21 mmHg during follow-up and the mean IOP at 9 months was 11.0 ± 3.1 mmHg with open angle configurations on UBM. Therefore, we are more inclined with the postulation that the initial optic nerve hypoperfusion from the phacomorphic attack induced cellular inflammatory cytokines and free radicals that led to progressive ganglion cell damage even after the initial phacomorphic angle closure resolved. It is also possible that there can be some initial optic nerve edema post attack that gradually subsides with time giving the impression of a “pseudo-thinning” but our first OCT was performed quite some time after the initial attack, at 3 months post operation, and by comparison with the contralateral eye, there was no significant difference in thickness, making this possibility less likely. Our study involving secondary angle closure reports RNFL thinning between 3 and 9 months post attack and our follow-up OCT is one of the longest reported in literature for acute angle closure.

Regarding the use of VF in the assessment post phacomorphic angle closure, a number of patients lacked the coordination to produce a reliable VF. Furthermore, the results produced at 9 months post attack failed to demonstrate any significant difference between the phacomorphic and contralateral eye but there was already significant RNFL thinning detected on OCT. There was also no correlation between the PSD on VF and the degree of RNFL thinning on OCT.

This study had its limitations. First, the assumption was made that both eyes had similar initial RNFL thickness and VCDR prior to the phacomorphic attack when using the contralateral eye as a proxy of the pre-phacomorphic state. This was because all of the cases presented to our center for the first time during the attack without any prior clinical data. It is possible that the documented RNFL thinning may be due to an underling normal tension glaucoma per se, therefore, in addition to comparing with the other eye, we also compared the progression of RNFL thinning in contralateral eye as well at 3–9 months and only found progression in the attack eye but not in the contralateral eye. Second, both ALPI and medical treatment was used to lower IOP but there is no evidence in the literature to support superiority of one treatment over the other and all IOP’s were lowered to 25 mmHg within 5 h of presentation. Our study further supports this evidence that the initial IOP lowering technique was not related to outcomes.

## Conclusion

In our prospective case series, patients with <5 days duration of phacomorphic angle closure had a mean Snellen vision of 0.4. An acute episode of phacomorphic angle closure can trigger an accelerated thinning of the average RNFL occurring between 3 and 9 months post phacomorphic attack despite normal IOP and open angles. The RNFL thinning was most prevalent in the superior and inferior quadrants as detected by OCT whilst visual field assessment failed to detect any significant differences between the attack and uninvolved eye.
